# Effect of Ultrasound‐Guided Hydrorelease of the Multifidus Muscle on Acute Low Back Pain

**DOI:** 10.1002/jum.15473

**Published:** 2020-08-25

**Authors:** Hirohito Kanamoto, Sumihisa Orita, Kazuhide Inage, Yasuhiro Shiga, Koki Abe, Yawara Eguchi, Seiji Ohtori

**Affiliations:** ^1^ Department of Orthopaedic Surgery Kanamoto Orthopaedic Clinic 760‐7 Matsunaga, Numazu, Shizuoka 410‐0874 Japan; ^2^ Department of Orthopaedic Surgery Graduate School of Medicine, Chiba University 1‐8‐1 Inohana, Chuo‐ku, Chiba 260‐8670 Japan

**Keywords:** low back pain, ultrasound‐guided hydrorelease, multifidus muscle, visual analog scale

## Abstract

**Objectives:**

To examine improvement in acute low back pain (LBP) using ultrasound‐guided hydrorelease of the multifidus muscle.

**Methods:**

This prognostic cohort study was conducted in a private clinic on samples of 75 patients with acute LBP diagnosed based on physical and imaging findings. Hydrorelease of the multifidus muscle was performed at the L4/5 level. The LBP visual analog scale (VAS) scores (cm) before and 5 minutes after hydrorelease were statistically evaluated. We defined improvement rate (%) as {LBP VAS scores (cm) immediately before hydrorelease – LBP VAS scores (cm) 5 minutes after hydrorelease} × 100 / LBP VAS scores (cm) immediately before hydrorelease and examined the correlation of the Heckmatt score and average age with the improvement rate.

**Results:**

LBP VAS scores (cm) before and 5 minutes after hydrorelease were 7.19 ± 1.01 (mean ± SD) and 2.85 ± 1.25, respectively (*p* < 0.05). No significant correlations were noted between the LBP improvement rate and the Heckmatt score or age. There were no gender variations in the improvement rate.

**Conclusions:**

Ultrasound‐guided hydrorelease of the multifidus muscle led to considerable LBP VAS score improvement at the outpatient level. The improvement rate showed no correlations with the Heckmatt score or age, and there were no significant gender variations in the improvement rate. Therefore, fatty degeneration of muscles and change in muscle echogenicity due to age and gender may not be associated with muscular LBP. These findings suggest that multifidus muscle hydrorelease could be useful in the diagnosis and treatment of acute LBP.

**Abbreviations**
CT: computed tomography
LBP: low back pain
MRI: magnetic resonance imaging
VAS: visual analog scale

## Introduction

Low back pain (LBP) is more common in men than in women. As per the Comprehensive Survey of Living Conditions in Japan (June 2013), most patients with LBP who visit outpatient orthopedic clinics are men. The cause of LBP may be multifactorial (e.g., related to muscles, fascia, intervertebral discs, facet joints, metastatic bone disease, degenerative bone disease, and nerve roots). LBP has various phases: acute, which lasts up to 4 weeks after onset; subacute, lasting for 4 to 12 weeks; and chronic, lasting for more than 12 weeks.^1^ Moreover, 85% of the cases present with nonspecific LBP, which is related to the muscles, fascia, intervertebral discs, facet joints, and nerve roots among others; diagnosis is often difficult.^2^, ^3^

Muscular LBP, a form of acute LBP, is experienced by 85% of people during their lifetime. Several muscles in the lumbar muscle group, including the multifidus, longissimus, iliocostalis, and quadratus lumborum muscles, can be cited as causes; however, the multifidus muscle, being greatly involved in the stability of the lumbar vertebra, plays an important role.^4^, ^5^, ^6^, ^7^

Although many reports have evaluated the limb trunk muscles for fatty degeneration, muscle mass, signs of infection, and other abnormal findings using computed tomography (CT) and magnetic resonance imaging (MRI),^8^, ^9^, ^10^, ^11^, ^12^ these tests are expensive, time‐consuming, and present only a static evaluation. Ultrasound examination is useful because it is rapid and noninvasive compared with other examinations.^7^, ^11^ It also presents a dynamic evaluation of the lesion site. Ultrasound is not inferior to CT or MRI in terms of diagnostic accuracy, depending on the site, but diagnostic accuracy requires optimization of the technique and good understanding of anatomic characteristics.^13^, ^14^, ^15^

Recently, abnormal findings have been observed using ultrasound examination. It has been reported that treatment by nerve root block and release to the surrounding soft tissue under ultrasound guidance is useful.^16^, ^17^, ^18^ The term “hydrorelease” is commonly used in Japan and is synonymous with the term “hydrodissection.” It improves the symptoms of strangulated perineural tissue.^19^, ^20^

In this study, we aimed to examine the improvement in acute LBP using ultrasound‐guided hydrorelease of the multifidus muscle and prove its usefulness in the diagnosis and treatment of acute LBP. Patients with acute back pain often experience atrophy of the lumbar multifidus muscle, limited to the segment of the affected side.^21^

Moreover, we examined the effects of multifidus hydrorelease in terms of age and gender, as well as on muscle degeneration with age.

## Materials and Methods

**Figure 1:**
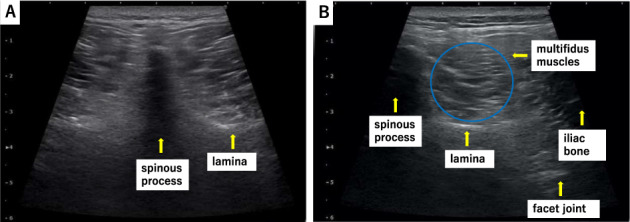
The multifidus muscle and surrounding anatomy at the L4/5 level in a healthy volunteer. **(A)** A probe is applied vertically to identify the spinous process on the axial view and **(B)** moved to the right or left to identify each tissue area of interest.

**Figure 2:**
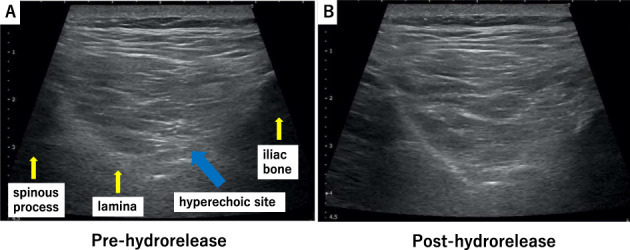
Hydrorelease of the multifidus muscle. Hydrorelease was performed using 7.0 mL of saline in the hyperechoic site of the multifidus muscle **(A)** pre‐hydrorelease; the blue arrow indicates the hyperechoic site. **(B)** Post‐hydrorelease, the hyperechoic site has disappeared.

**Figure 3:**
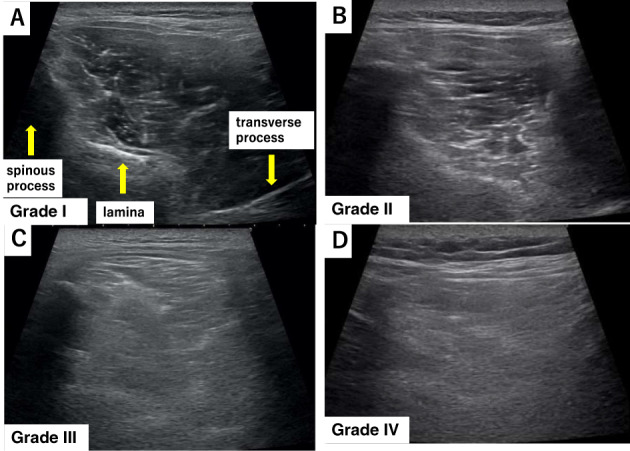
Heckmatt score: The classification of muscle echogenicity. **(A)** normal echogenicity, **(B)** increased muscle echogenicity with normal bone reflection, **(C)** increased muscle echogenicity with reduced bone reflection, and **(D)** markedly increased muscle echogenicity and loss of bone reflection.

This prognostic cohort study included 75 patients with acute LBP (average age: 54.8 ± 14.8 years; 54 men and 21 women) who were diagnosed based on physical and imaging findings and were suspected of having muscular LBP, primarily owing to a load on the waist without bone and joint abnormalities. We excluded conditions requiring urgent treatment such as fractures, infectious spondylitis, cancer metastasis, and the cauda equina syndrome. Ethical approval was provided by the institutional review board of the University of Chiba, Japan (IRB_2894). All study participants provided informed consent. Jacoby's line was palpated to identify the multifidus muscle at the L4/5 level. Ultrasound was performed using a 4–18 MHz probe real‐time linear array scanner (SNiBLE, Konica Minolta, Tokyo, Japan). The probe was applied vertically to identify the spinous process on the axial view carefully, never tilting it back and forth, and was moved to the right or left to identify each tissue area of interest (Figure 1). Ultrasound‐guided hydrorelease was performed using 7.0 mL saline injected at a moderate rate with the needle (23G, 60 mm) directed at the site with hyperechoic changes in the multifidus muscle (Figure 2). No local anesthesia or analgesia was induced before the procedure; medication and physical therapy were added after hydrorelease. The site with hyperechoic changes was identified by carefully comparing both sides of the multifidus muscle. Hydrorelease was performed on one or both sides of the multifidus muscle, depending on the site of pain. The Heckmatt score was used for evaluating muscle degeneration. The grading was defined as follows: grade I: normal muscle echogenicity; grade II: increased muscle echogenicity with normal bone reflection; grade III: increased muscle echogenicity with reduced bone reflection; and grade IV: markedly increased muscle echogenicity with loss of bone reflection (Fig. 3).^11^, ^22^, ^23^ The increase in the grade correlated with the amount of muscle fatty degeneration; the bone images were obscured by acoustic shadows from the overlying muscle with a denser echotexture. Muscle fibrosis occurs with age, even in normal conditions, and the brightness increases slightly. Therefore, it is necessary to judge normal and abnormal conditions with respect to the patient's age.

The LBP visual analog scale (VAS) scores (cm) before and 5 minutes after hydrorelease were statistically evaluated. After hydrorelease, additional treatments such as medication and physical therapy were administered. Therefore, the aftereffects of hydrorelease at 30 minutes and 24 hours could not be accurately evaluated. Therefore, we determined that 5 minutes was appropriate for a single evaluation without additional treatment. We defined the improvement rate (%) as {LBP VAS scores (cm) immediately before hydrorelease – LBP VAS scores (cm) 5 minutes after hydrorelease} × 100 / LBP VAS scores (cm) immediately before hydrorelease. We examined the correlation of the Heckmatt score and average age with the improvement rate. For statistical analysis, Pearson's correlation and Student's *t* test analyses were performed using R version 3.2.1 (www.R-project.org), and differences were considered statistically significant at *p‐*values <0.05.

## Results

**Figure 4:**
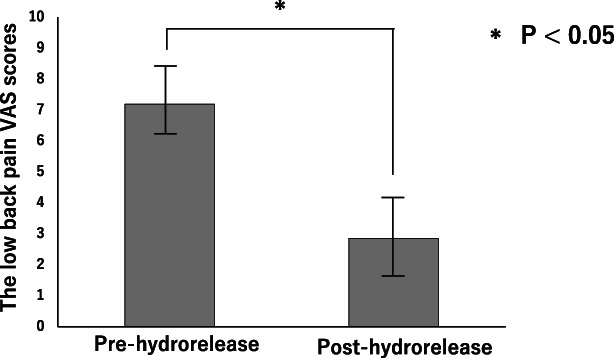
Changes in the low back pain (LBP) visual analog scale (VAS) scores after hydrorelease of the multifidus muscle. The average (mean ± SD) LBP VAS scores (cm) before and 5 minutes after hydrorelease were 7.19 ± 1.01 and 2.85 ± 1.25, respectively (*p* < 0.05).

**Figure 5:**
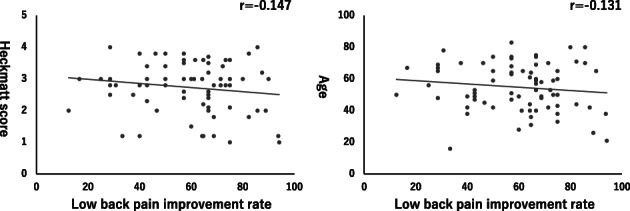
Relationship of the low back pain (LBP) improvement rate with the Heckmatt score and age. The LBP improvement rate did not correlate with the Heckmatt score (r = −0.147) and age (r = −0.131).

**Figure 6:**
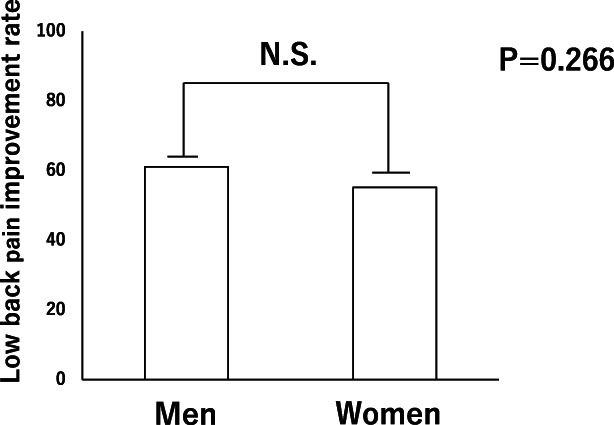
Rate of low back pain improvement in men and women. There was no significant difference in the rate of improvement between men and women (*p* = 0.266).

The average (mean ± SD) LBP VAS scores (cm) before and 5 minutes after hydrorelease were 7.19 ± 1.01 and 2.85 ± 1.25, respectively (*p* < 0.05) (Figure 4). There was no correlation between the LBP improvement rate and Heckmatt score (r = −0.147) and between the LBP improvement rate and age (r = −0.131) (Figure 5). There was also no significant difference in the rate of improvement between men and women (*p* = 0.266) (Figure 6).

## Discussion

LBP is refractory and may often be a major obstacle to activities of daily living. In this study, the considerable improvement in LBP VAS scores was probably a result of ultrasound‐guided hydrorelease of the multifidus muscle performed at the outpatient level; hence, it seems worthy of consideration.

When LBP occurs, the dorsal muscle group, particularly the multifidus muscle, is degenerated.^24^ A deficit in the multifidus muscle, which does not resolve spontaneously on the resolution of painful symptoms and resumption of normal activities, has been identified in acute LBP patients.^25^ However, there is a remodeling process for damaged muscle fibers.^26^ The site of hyperechoic change in the multifidus muscle may indicate the site for remodeling of the damaged muscle (muscle fibers).

In myofascial pain, relieving pain by hydrorelease of the fascia is based physiologically on the presence of pain receptors in the fascia.^27^, ^28^ Stimulation of tissues, such as the muscle fibers, facet joint capsule, nerve root, and annulus fibrosus of the disc, may induce an increase in the sensitivity of the lumbar fascia dominated by the same nerve branch of the spinal cord.^29^, ^30^, ^31^, ^32^ Therefore, releasing the nerve from the multifidus muscle may improve LBP caused by nociceptors in the fascia. This theory is consistent with the fact that ultrasound‐guided hydrorelease of the multifidus muscle is effective, even in older adults and patients with a high Heckmatt score.

It is reportedly safer to dilute the local anesthetic with 5% dextrose than to use the local anesthetic alone.^33^ It has also been reported that saline alone is more effective than the combination of mepivacaine and saline for eliminating pain when releasing fascia.^34^ Hence, saline was selected in this study, considering its safety and pain relief characteristics.

In this study, the improvement rate did not correlate with the Heckmatt score or age, and there were no significant differences between men and women; therefore, it may be concluded that fatty degeneration of muscles and muscle echogenicity changes due to age and gender are not the factors associated with muscular LBP. In the non‐improved cases, it is possible that the cause of acute LBP is not only muscular but may involve factors such as intervertebral disc, facet joint, and lumbar spine degeneration.

Good results were obtained in acute LBP using hydrorelease of the multifidus muscle.

This study has several limitations. First, hydrorelease of the multifidus muscle was only performed at the L4/5 level; it is important to examine other levels. Second, the study had no control group; ie, the group that underwent hydrorelease was not compared to patients with LBP that did not undergo hydrorelease. Third, the VAS uses some subjective evaluations, including a psychological element and a physical stressor; therefore, other evaluation methods should also be considered.

We report good results for acute LBP relief using ultrasound‐guided hydrorelease of the multifidus muscle. Our findings suggest that muscle fatty degeneration and muscle echogenicity changes based on age and gender do not affect muscular LBP. It is therefore suggested that ultrasound‐guided hydrorelease of the multifidus muscle may be useful in the diagnosis and treatment of acute LBP.

## Competing interests

The authors declare that they have no competing interests.

## References

[1] Abenhaim L , Rossignol M , Valat JP , et al. The role of activity in the therapeutic management of back pain. Report of the International Paris Task Force on Back Pain. Spine 2000; 25:1S–33S. 10707404 10.1097/00007632-200002151-00001

[2] Friedman BW , Chilstrom M , Bijur PE , Gallagher EJ . Diagnostic testing and treatment of low back pain in United States emergency departments: a national perspective. Spine 2010; 35:E1406–E1411. 21030902 10.1097/BRS.0b013e3181d952a5 PMC2982879

[3] van Tulder M , Becker A , Bekkering T , et al. Chapter 3. European guidelines for the management of acute nonspecific low back pain in primary care. Eur Spine J 2006; 151:S169–S191. 10.1007/s00586-006-1071-2 PMC3454540 16550447

[4] Ward SR , Kim CW , Eng CM , et al. Architectural analysis and intraoperative measurements demonstrate the unique design of the multifidus muscle for lumbar spine stability. J Bone Joint Surg Am 2009; 91:176–185. 19122093 10.2106/JBJS.G.01311 PMC2663324

[5] MacDonald D , Moseley GL , Hodges PW . Why do some patients keep hurting their back? Evidence of ongoing back muscle dysfunction during remission from recurrent back pain. Pain 2009; 142:183–188. 19186001 10.1016/j.pain.2008.12.002

[6] Moseley GL , Hodges PW , Gandevia SC . Deep and superficial fibers of the lumbar multifidus muscle are differentially active during voluntary arm movements. Spine 2002; 27:E29–E36. 11805677 10.1097/00007632-200201150-00013

[7] Hebert JJ , Koppenhaver SL , Teyhen DS , Walker BF , Fritz JM . The evaluation of lumbar multifidus muscle function via palpation: reliability and validity of a new clinical test. Spine J 2015; 15:1196–1202. 24314767 10.1016/j.spinee.2013.08.056 PMC3976459

[8] Danneels LA , Vanderstraeten GG , Cambier DC , Witvrouw EE , De Cuyper HJ . CT imaging of trunk muscles in chronic low back pain patients and healthy control subjects. Eur Spine J 2000; 9:266–272. 11261613 10.1007/s005860000190 PMC3611341

[9] Keller A , Johansen JG , Hellesnes J , Brox JI . Predictors of isokinetic back muscle strength in patients with low back pain. Spine 1999; 24:275–280. 10025023 10.1097/00007632-199902010-00016

[10] Peltonen JE , Taimela S , Erkintalo M , Salminen JJ , Oksanen A , Kujala UM . Back extensor and psoas muscle cross‐sectional area, prior physical training, and trunk muscle strength – a longitudinal study in adolescent girls. Eur J Appl Physiol Occup Physiol 1998; 77:66–71. 9459523 10.1007/s004210050301

[11] Zaidman CM , Malkus EC , Siener C , Florence J , Pestronk A , Al‐Lozi M . Qualitative and quantitative skeletal muscle ultrasound in late‐onset acid maltase deficiency. Muscle Nerve 2011; 44:418–423. 21755514 10.1002/mus.22088 PMC3193541

[12] Ploumis A , Michailidis N , Christodoulou P , Kalaitzoglou I , Gouvas G , Beris A . Ipsilateral atrophy of paraspinal and psoas muscle in unilateral back pain patients with monosegmental degenerative disc disease. Br J Radiol 2011; 84:709–713. 21081573 10.1259/bjr/58136533 PMC3473439

[13] Seibold CJ , Mallisee TA , Erickson SJ , Boynton MD , Raasch WG , Timins ME . Rotator cuff: evaluation with US and MR Imaging. Radiographics 1999; 19:685–705. 10336198 10.1148/radiographics.19.3.g99ma03685

[14] Yang L , Ran H . Extracranial vertebral artery dissection: findings and advantages of ultrasonography. Medicine (Baltimore) 2018 Mar; 97:e0067. 10.1097/MD.0000000000010067. 29489668 PMC5851752

[15] Jacobson JA . Musculoskeletal ultrasound: focused impact on MRI. AJR Am J Roentgenol 2009; 193:619–627. 19696273 10.2214/AJR.09.2841

[16] Yoshida T , Nakamoto T , Kamibayashi T . Ultrasound‐guided obturator nerve block: a focused review on anatomy and updated techniques. BioMed Res Int 2017; 2017:7023750. 28280738 10.1155/2017/7023750 PMC5322453

[17] Gray AT . Ultrasound‐guided regional anesthesia: current state of the art. Anesthesiology 2006; 104:368–373. 16436858 10.1097/00000542-200602000-00024

[18] Akkaya T , Ozturk E , Comert A , et al. Ultrasound‐guided obturator nerve block: a sonoanatomic study of a new methodologic approach. Anesth Analg 2009; 108:1037–1041. 19224822 10.1213/ane.0b013e3181966f03

[19] Lam SKH , Reeves KD , Cheng AL . Transition from deep regional blocks toward deep nerve hydrodissection in the upper body and torso: method description and results from a retrospective chart review of the analgesic effect of 5% dextrose water as the primary hydrodissection injectate to enhance safety. BioMed Res Int 2017; 2017:7920438. 29226148 10.1155/2017/7920438 PMC5684526

[20] Cass SP . Ultrasound‐guided nerve hydrodissection: what is it? A review of the literature. Curr Sports Med Rep 2016; 15:20–22. 26745165 10.1249/JSR.0000000000000226

[21] Hides JA , Stokes MJ , Saide M , Jull GA , Cooper DH . Evidence of lumbar multifidus muscle wasting ipsilateral to symptoms in patients with acute/subacute low back pain. Spine 1994; 19:165–172. 8153825 10.1097/00007632-199401001-00009

[22] Grimm A , Teschner U , Porzelius C , et al. Muscle ultrasound for early assessment of critical illness neuromyopathy in severe sepsis. Crit Care 2013; 17:R227. 24499688 10.1186/cc13050 PMC4057413

[23] Annetta MG , Pittiruti M , Silvestri D , et al. Ultrasound assessment of rectus femoris and anterior tibialis muscles in young trauma patients. Ann Intensive Care 2017; 7:104. 28986861 10.1186/s13613-017-0326-x PMC5630542

[24] Wagner H , Anders C , Puta C , et al. Musculoskeletal support of lumbar spine stability. Pathophysiology 2005; 12:257–265. 16239098 10.1016/j.pathophys.2005.09.007

[25] Hides JA , Jull GA , Richardson CA . Long‐term effects of specific stabilizing exercises for first‐episode low back pain. Spine 2001; 26:E243–E248. 11389408 10.1097/00007632-200106010-00004

[26] Jakobsen JR , Jakobsen NR , Mackey AL , Koch M , Kjaer M , Krogsgaard MR . Remodeling of muscle fibers approaching the human myotendinous junction. Scand J Med Sci Sports 2018; 28:1859–1865. 29672952 10.1111/sms.13196

[27] Wilke J , Schleip R , Klingler W , Stecco C . The lumbodorsal fascia as a potential source of low back pain: a narrative review. BioMed Res Int 2017; 2017:5349620. 28584816 10.1155/2017/5349620 PMC5444000

[28] Tesarz J , Hoheisel U , Wiedenhöfer B , Mense S . Sensory innervation of the thoracolumbar fascia in rats and humans. Neuroscience 2011; 194:302–308. 21839150 10.1016/j.neuroscience.2011.07.066

[29] Langevin HM , Sherman KJ . Pathophysiological model for chronic low back pain integrating connective tissue and nervous system mechanisms. Med Hypotheses 2007; 68:74–80. 16919887 10.1016/j.mehy.2006.06.033

[30] Umimura T , Miyagi M , Ishikawa T , et al. Investigation of dichotomizing sensory nerve fibers projecting to the lumbar multifidus muscles and intervertebral disc or facet joint or sacroiliac joint in rats. Spine 2012; 37:557–562. 21697769 10.1097/BRS.0b013e3182293178

[31] Wakai K , Ohtori S , Yamashita M , et al. Primary sensory neurons with dichotomizing axons projecting to the facet joint and the low back muscle in rats. J Orthop Sci 2010; 15:402–406. 20559809 10.1007/s00776-010-1465-1

[32] Sameda H , Takahashi Y , Takahashi K , Chiba T , Ohtori S , Moriya H . Primary sensory neurons with dichotomizing axons projecting to the facet joint and the sciatic nerve in rats. Spine 2001; 26:1105–1109. 11413420 10.1097/00007632-200105150-00003

[33] Dufour E , Donat N , Jaziri S , et al. Ultrasound‐guided perineural circumferential median nerve block with and without prior dextrose 5% hydrodissection: a prospective randomized double‐blinded noninferiority trial. Anesth Analg 2012; 115:728–733. 22745114 10.1213/ANE.0b013e31825fa37d

[34] Frost FA , Jessen B , Siggaard‐Andersen J . A control, double‐blind comparison of mepivacaine injection versus saline injection for myofascial pain. Lancet 1980; 1:499–500. 6102230 10.1016/s0140-6736(80)92761-0

